# Incidence of Thrombotic Events and Outcomes in COVID-19 Patients Admitted to Intensive Care Units

**DOI:** 10.7759/cureus.11079

**Published:** 2020-10-21

**Authors:** Akshay Avula, Krishna Nalleballe, Sudhamshi Toom, Suman Siddamreddy, Dhineshreddy Gurala, Nakul Katyal, Srikanth Maddika, Abhishek D Polavarapu, Rohan Sharma, Sanjeeva Onteddu

**Affiliations:** 1 Internal Medicine, Northwell Health - Staten Island University Hospital, Staten Island, USA; 2 Neurology/Stroke, University of Arkansas for Medical Sciences, Little Rock, USA; 3 Hematology and Medical Oncology, Maimonides Medical Center, Brooklyn, USA; 4 Internal Medicine, Baptist Health Center, Little Rock, USA; 5 Neurology, University of Missouri, Columbia, USA; 6 Internal Medicine, St. Barnabas Hospital Health System, Bronx, USA; 7 Gastroenterology and Hepatology, Northwell Health - Staten Island University Hospital, Staten Island, USA

**Keywords:** covid-19, thrombosis, icu, mortality, outcomes

## Abstract

Introduction

While coronavirus disease 2019 (COVID-19) mostly causes respiratory illnesses, emerging evidence has shown that patients with severe COVID-19 can develop complications like venous thromboembolism (VTE) and arterial thrombosis as well. The incidence of thrombosis among critically ill patients in the literature has been highly variable, ranging from 25 to 69%. Similarly, reported mortality among critically ill patients has been highly variable too, and it has ranged from 30 to 97%. In this study, we analyzed data from a large database to address the incidence, the risk factors leading to thrombotic complications, and mortality rates among COVID-19 patients.

Material and methods

Data were obtained from TriNetX (TriNetX, Inc., Cambridge, MA), a multinational clinical research platform that collects medical records from 42 healthcare organizations (HCOs). All nominal data were compared using the chi-squared test. Alpha of <0.05 was considered statistically significant. We used Benjamini-Hochberg correction with a false discovery rate of 0.1 to correct for multiple comparisons.

Results

We identified 18,652 COVID-19-positive patients, with a median age of 50.7 years [interquartile range (IQR): 31.8-69.6]; among them, 51.8% (9,672) were males and 48.2% (8,951) were females. Of these patients, 630 [3.37%; median age: 61 years (IQR: 44.9-77.1)] were critically ill, requiring intensive care unit (ICU) care within one month of their diagnosis. Men were over-represented among the ICU patients when compared to women (3.7% vs 3%, p=0.009, Χ^2^=6.66). African Americans were over-represented among the ICU patients when compared to Caucasians (8.5% vs 4%, p<0.0001, Χ^2^=76.65). Older patients, i.e., 65 years and older, were over-represented in the ICU compared to patients aged 18-64 years (6.8% vs 2.5%, p<0.0001, Χ^2^=121.43).

The cumulative incidence of thrombotic events in the ICU population was 20.4% (129/630). Thrombotic events were significantly more common in patients who were 65 years and older when compared to patients in the age group of 18-64 years (24.6% vs 17.31%, p=0.02, Χ^2^=5.38). Mortality among ICU patients was higher in those who were 65 years and older when compared to the age group of 18-64 years (31.9% vs 17.3% p=0.0003, Χ^2^=18.41). The overall mortality in the study population was higher in patients who were 65 years and older when compared to patients aged 18-64 years (18.55% vs 1.4%, p<0.0001, Χ^2^=1915).

Conclusions

Among COVID-19 patients, men, African Americans, and people who are 65 years and older are more likely to have severe disease and require ICU level of care. Patients who are 65 years and older are more likely to have thrombotic events, myocardial infarction (MI), and stroke. Overall mortality and ICU mortality are higher among COVID-19 patients who are 65 years and older.

## Introduction

Coronavirus disease 2019 (COVID-19) was first reported in the city of Wuhan, China in December 2019 and has quickly progressed to become a global pandemic by March 2020. COVID-19 is a heterogeneous disease entity caused by the novel coronavirus 2019, which is now called severe acute respiratory syndrome coronavirus 2 (SARS-CoV-2) [1]. While COVID-19 mostly causes respiratory illnesses, emerging evidence has shown that patients with severe COVID-19 can develop complications like coagulopathy and disseminated intravascular coagulation (DIC) leading to venous thromboembolism (VTE) along with arterial thrombosis [2,3]. Thromboembolism among patients hospitalized with COVID-19 has been reported to be in the range of 10-25% in previous studies [4,5]. Critically ill patients in intensive care units (ICUs) are at an increased risk due to immobilization, insertion of central venous catheters alongside severe inflammation such as cytokine storm syndrome [6].

Age and coagulopathy have been identified as independent risk factors for thrombotic complications among COVID-19 patients [7]. Thromboembolism has been associated with a worse prognosis [8,9]. Incidence of venous and arterial thrombosis has been reported to range between 25-69% among critically ill patients in the ICU [10]. However, these studies are limited by a small patient population, and heterogeneity of the study design, potentially leading to bias.

The reported case mortality rate among COVID-19 patients admitted to the ICU has been highly variable, ranging between 30-97% in those requiring mechanical ventilation [11,12,13]. This high variability is due to the way data is reported, the difference in the completion of the data, and variable patient cohorts, etc.

Precise knowledge about the incidence, the risk factors leading to thrombotic complications, and mortality are critical to treating these patients appropriately. In this study, we analyze data from a large database comprising data of more than 65,000 COVID-19-positive patients to address these issues. In addition, we analyze the mortality trend in various subsets of patients with COVID-19.

## Materials and methods

The data were obtained from TriNetX (TriNetX, Inc., Cambridge, MA), a multinational collaborative clinical research platform that collects real-time medical records from 42 healthcare organizations (HCOs) globally. Typical HCOs are large academic health centers and their affiliates. TriNetX’s “COVID-19 Research Network” represents a large COVID-19 dataset. Data obtained from TriNetX’s platform were de-identified and updated in real-time. TriNetX does not allow data download. Data extraction and analysis were done on the TriNetX browser on June 12, 2020. The data on ICU admissions were determined based on critical care billing codes within one month of diagnosis of COVID-19. The primary outcome of interest was the incidence of thrombotic events in the ICU population. Secondary outcomes of the study were as follows: (1) comparison of the incidence of thrombotic events among ICU patients aged 18-64 years vs those who were 65 years and older, (2) comparison of mortality among ICU patients aged 18-64 years old vs those who were 65 years and older.

We queried the dataset for adults aged >18 years of age with laboratory-confirmed reverse transcription-polymerase chain reaction (RT-PCR) positive COVID-19 tests from January 20, 2020, to June 12, 2020 (study period). We then identified patients admitted to the ICU within one month of their diagnosis. Demographic information was analyzed for age, gender, and race; we then divided the study populations into two groups based on age: patients aged 18-64 years and those aged 65 years and above. The average age difference was compared between the 18-64-year group and the group of patients aged 65 years and above using pooled variance t-test since the standard deviations for the two groups were similar. The ratio of ICU admissions was compared in COVID-19 patients between men/women and African Americans/Caucasians. The ratio of total deaths was compared between the 18-64-year group and the group of patients aged 65 years and above, as well as among those admitted to the ICU. Also, the ratio of clinical outcomes such as thrombotic events [defined as pulmonary embolism (PE), deep vein thrombosis (DVT), myocardial infarction (MI), or strokes], mortality, etc., between the two groups was also compared. All nominal data were compared using the chi-squared test. Alpha of 0.05 was considered statistically significant. We used Benjamini-Hochberg correction with a false discovery rate of 0.1 to correct for multiple comparisons.

## Results

Demographics

During the study period, we identified 18,652 COVID-19-positive patients, with a median age of 50.7 years [interquartile range (IQR): 31.8-69.6]; among them, 51.8% (9,672) were males and 48.2% (8,951) were females. The racial distribution of the patients was as follows: 34% (6,354) Caucasian, 15.4% (2,891) African American, 1.5% (294) Asian, and 48.9% (9,113) other/unknown. Among the study population, 76.05% (14,184) were 18-64 years of age and 23.95% (4,468) were 65 years and older.

Among these patients, 630 [3.37%; median age: 61 years (IQR, 44.9-77.1)] patients were critically ill, requiring ICU care within one month of diagnosis. Men were over-represented in the ICU as compared to women (3.7% vs 3%, p=0.009, Χ^2^=6.66). The racial distribution of patients admitted to the ICU was as follows: 41.1% (259) Caucasian, 39.2% (247) African American, 2.3% (15) Asian, and 20.9% (132) other/unknown. African Americans were over-represented in the ICU as compared to Caucasians (8.5% vs 4%, p<0.0001, Χ^2^=76.65). Among the ICU population, 56.8% (358) were 18-64 years of age (median: 49.8; IQR: 38.3-61.3), and 43.1% were 65 years and older (median: 75.3, IQR: 67.6-82.9). There was no statistical difference in mean age between those admitted to ICU as compared to those not requiring ICU admission (p=0.2). The difference in the mean age was statistically significant between the 18-64-year-olds and those who were 65 years and older (difference in mean age=25.5 +/-19.6, p=0.005). Among the study population, older patients, i.e., 65 years and older, were over-represented in the ICU when compared to patients aged 18-64 years (6.8% vs 2.5%, p<0.0001, Χ^2^=121.43) (Table 1).

**Table 1:** Demographic details of COVID-19 patients *Pooled variance t-test; **Pearson’s chi-squared test ICU: intensive care unit

Variables	Overall cohort	ICU patients	P-value, X^2^
Number of patients	18,652	630	
Median age (years)	50.7 +/-18.9	61 +/-16.1	0.2*
Sex			
Men	9,672	359	
Women	8,951	271	0.009, 6.66**
Race			
White	6,354	259	
African American	2,891	247	<0.0001, 76.65**
Asian	294	15	0.38, 0.75**
Unknown/other	9,113	132	

Clinical outcomes in the ICU population

The cumulative incidence of thrombotic events in the ICU population was 20.4% (129/630). Thrombotic events were significantly more common in patients who were 65 years and older compared to patients in the age group of 18-64 years (24.6% vs 17.31%, p=0.02, Χ^2^=5.38). The cumulative incidence of VTE, including PE and DVT, was 8.4%. The incidence of VTE was higher in the 18-64 age group compared to those who were 65 years and older, but the difference was not statistically significant (p=0.4, Χ^2^=0.7). The cumulative incidence of MI was 7.6%. The incidence of MI was significantly higher in patients who were 65 years and older compared to those aged 18-64 years (9.9% vs 5.8%, p=0.02, Χ^2^=5.25). The cumulative incidence of stroke was 6.3%. Stroke was more common in patients who were 65 years and older compared to those aged 18-64 years (10.29% vs 3.3%, p=0.0004, Χ^2^=12.53). Mortality among ICU patients was higher in those who were 65 years and older when compared to the age group of 18-64 years (31.9% vs 17.3% p=0.0003, Χ2=18.41). The overall mortality in the study population was higher in patients who were 65 years and older when compared to patients aged 18-64 years (18.55% vs 1.4%, p<0.0001, Χ2=1915) (Table 2).

**Table 2:** Clinical outcomes in ICU patients *Pooled variance t-test; **Pearson’s Chi-square test ICU: intensive care unit; PE: pulmonary embolism; DVT: deep vein thrombosis; MI: myocardial infarction

Variables	18-64 years	65 years and older	P-value, Χ^2^
Total number of patients	14,184	4,468	
Number of patients in ICU	358	272	<0.0001, 121.43**
Mean age (years)	49.8 +/-11.5	75.3 +/-7.6	0.005*/(Diff=25.5 +/-19.6)
Clinical outcomes in ICU patients			
All thrombotic events	62	67	0.02, 5.38**
PE + DVT	33	20	0.4, 0.7**
MI	21	27	0.02, 5.25**
Stroke	12	28	0.0004, 12.53**
Total deaths	200	829	<0.0001, 1915**
Deaths in ICU	62	87	0.0003, 18.41**

## Discussion

COVID-19 is associated with variable clinical presentations, ranging from asymptomatic disease to severe disease requiring ICU care [14]. It is imperative to identify the high-risk population as it will help us to allocate the already scarce resources to them on a priority basis during this pandemic. The median age of ICU patients with COVID-19 in our analysis (61) was pretty close to the median age of patients (62.4) as reported in a recent meta-analysis [15].

Previous studies have shown that hospitalization, ICU admissions, and death are significantly higher in the black population than in the white cohort [16]. In our analysis, the number of black patients with COVID-19 admitted to hospitals and ICU was less compared to whites. This can be attributed to the fact that the race of a large percentage of the population was not clearly mentioned in the database, which might have skewed the demographic data. Despite this limitation, although the black population accounted for only 15% of total positive cases, 40% of the patients admitted to the ICU were black.

Emerging evidence suggests that men with COVID-19 have more hospitalizations, ICU admissions, and composite deaths in various countries [17]. Our analysis further supports the finding that the percentage of men among COVID-19 patients admitted to hospitals and ICU is significantly more than that of women.

Several factors, such as the definition of the severity of illness and ICU capability of the healthcare system, affect the number of ICU admissions. In a summary report from the Chinese Center for Disease Control and Prevention, 5% of COVID-19 patients got admitted to ICU, out of which 49% died [14]. In our analysis, patients who were 18 years and older constituted 3.37% (630/18,652) of those admitted to ICU. Patients who were 65 years and older (6.08%) were overrepresented in the ICU compared to the age group of 18-64 years (2.52%). Similarly, among patients admitted to the ICU, higher mortality (31.9%) was recorded in patients who were 65 years and older when compared to the age group of 18-64 years (17.3%). These results are consistent with those of previous studies, which have also reported significantly higher mortality rates among patients who are 65 years and older [11,18]. This can be attributed to a higher incidence of comorbidities in patients who are 65 years and older. As seen in other respiratory outbreaks, comorbidities such as chronic obstructive pulmonary disease (COPD), diabetes, hypertension, and malignancies predispose patients to adverse clinical outcomes [19].

Immobilization, excessive inflammation, and DIC are possible causes of the increased incidence of VTE in severe COVID-19 patients. A few autopsy findings have shown diffuse microthrombi in multiple organs without any virus infiltrates [7,20]. Immunoglobulin A (IgA) antiphospholipid antibodies have been described in patients with severe COVID-19, raising the question of whether there is any role of complement blockade in severe COVID-19 patients [21]. As reported in recent studies, several COVID-19 patients with severe acute respiratory distress syndrome (ARDS) can develop severe thrombotic complications despite being treated with prophylactic anticoagulation [22,23]. A higher incidence of thrombosis in SARS-CoV compared to H1N1 has been reported [24]. Consistent with the findings of other studies, older age has been shown to be a risk factor for thrombotic patients in our analysis as well. It holds true when thrombotic complications are considered as a cumulative complication.

Limitations

The major limitation of our study was that it was a retrospective database study, and hence lacked randomization. A large proportion of patients did not have their race reported. ICU admission data were determined by critical care billing codes within one month of diagnosis. However, not all critically ill patients could have been billed as per critical care billing codes, which could have led to under-reporting of the critically ill patient population. Lastly, due to the limitations of the software that the database uses, a multivariate analysis could not be performed to rule out confounding factors. Despite these limitations, we believe that the findings of our study would be valuable to the clinical and research community as these would help them to determine real-world outcomes related to the treatment of COVID-19 patients.

Interestingly, in our analysis, ICU patients who were 65 and older had a significantly higher percentage of MI, stroke, total composite deaths, and deaths, but not VTE. The lower incidence of VTE in our study could be attributed to under-testing and under-reporting. In patients who were 65 years and older, this could be attributed to a higher incidence of comorbidities like diabetes, malignancies, hypertension, and prior cardiovascular diseases.

## Conclusions

Based on our study of COVID-19 patients, men, African Americans, and those who are 65 years and older are more likely to have severe disease and require ICU level of care. Patients who are 65 years and older are more likely to have thrombotic events, MI, and stroke. Overall mortality and ICU mortality rates are higher among COVID-19 patients who are 65 years and older.
